# Podophyllotoxin-polyacrylic acid conjugate micelles: improved anticancer efficacy against multidrug-resistant breast cancer

**DOI:** 10.1186/s43046-020-00053-1

**Published:** 2020-11-16

**Authors:** Popat S. Kumbhar, Asmita M. Sakate, Onkar B. Patil, Arehalli S. Manjappa, John I. Disouza

**Affiliations:** Department of Pharmaceutics, Tatyasaheb Kore College of Pharmacy, Warananagar, Panhala, Kolhapur, Maharashtra 416113 India

**Keywords:** Podophyllotoxin prodrug, PAA, Hemolysis, In vitro release, Cytotoxicity

## Abstract

**Background:**

Podophyllotoxin (PPT) is a naturally occurring compound obtained from the roots of Podophyllum species, indicated for a variety of malignant tumors such as breast, lung, and liver tumors. This toxic polyphenol (PPT) exhibited significant activity against P-glycoprotein (P-gp) mediated multidrug-resistant (MDR) cancer cells. However, extremely poor water solubility, a narrow therapeutic window, and high toxicity have greatly restricted the clinical uses of PPT. Therefore, the present research was aimed to synthesize the water-soluble ester prodrug of PPT with polyacrylic acid (PAA), a water-soluble polymer by Steglich esterification reaction, and to screen it for assay, solubility, in vitro hemolysis, in vitro release, and in vitro anticancer activity.

**Results:**

The Fourier transform infrared (FTIR) and nuclear magnetic resonance (NMR) spectroscopy results revealed the successful synthesis of podophyllotoxin-polyacrylic acid conjugate (PPC). The assay and saturation solubility of the prodrug is found to be 64.01 ± 4.5% and 1.39 ± 0.05 mg/mL (PPT equivalent) respectively. The PPC showed CMC (critical micelle concentration) of 0.430 mg/mL in distilled water at room temperature. The PPC micelles showed a mean particle size of 215 ± 11 nm with polydispersity index (PDI) of 0.193 ± 0.006. Further, the transmission electron microscope (TEM) results confirmed the self-assembling character of PPC into micelles. The PPC caused significantly less hemolysis (18.6 ± 2.9%) than plain PPT solution. Also, it demonstrated significantly (*p* < 0.01) higher in vitro cytotoxicity against both sensitive as well as resistance human breast cancer cells (MCF-7 and MDA MB-231) after 48 h of treatment.

**Conclusion:**

The obtained study results clearly revealed the notable in vitro anticancer activity of PPT following its esterification with PAA. However, further in vivo studies are needed to ascertain its efficacy against a variety of cancers.

## Background

Cancer or malignancy is a heterogeneous disease characterized by abnormal cell mitosis, and is a serious health concern around the world. Cancer predominance and mortality are expanding year by year and creating a heavier burden globally [1].

Chemotherapy is the most preferred among the available treatment strategies and has been proven to be effective in clinics. But, the multidrug resistance (MDR) is one of the challenges in the efficient treatment of cancers. A toxic polyphenol podophyllotoxin (PPT) was obtained from the roots of plants from the genus Podophyllum [2] and can be used to treat cancers like breast, lung, and liver cancer and it acts by blocking cell division [3, 4]. In earlier research papers, it is reported that PPT is capable to kill effectively the MDR (P-gp mediated) cancer cells and therefore used to treat a variety of MDR tumors efficiently [5–8]. However, the clinical applications of PPT are significantly restricted due to its enormously poor water solubility, narrow therapeutic window, and high toxicity [9]. Also, chemotherapy of PPT is allied with serious side effects owing to non-specific distribution and lack of selectivity.

Therefore, to beat the conventional chemotherapeutic drug-related issues, various novel nanoformulations such as nanoparticles, nanocapsules, liposomes, polymer-drug conjugates, and polymeric micelles have been proposed. Currently, the polymer-drug conjugate is a chiefly used strategy wherein the polymers are used to modify the chemical structure of the drug without changing the original medicinal use of the drug [10]. In this strategy, sometimes the hydrophobic drug is conjugated with a hydrophilic polymer or vice versa. These polymer-drug conjugates show improved drug entrapment in the nanoparticles, biopharmaceutical properties, selectivity to tumor cells, tumor cell sensitivity, and in vivo stability [11]. Some researchers have developed a prodrug for different drugs like paclitaxel, docetaxel, and used alone or with different delivery carriers [12–14]. Moreover, Greenwald and co-workers have observed that PPT can be faster released from polymer-drug conjugate prodrug and has stronger inhibition of cell growth [15].

Polyacrylic acid (PAA) is one of the highly hydrophilic, absorbent, and biodegradable polymers with a number of industrial and biomedical applications. It is used widely in modified-release tablets, oral suspensions, and bioadhesive mean for mucosal and oral contact applications [16]. This copolymer is considered as a pharmaceutically safe. Carboxylic acid groups of PAA facilitate further modifications as well as assist binding of small molecules (drugs) and large molecules (biologics) in mild conditions with no structural deterioration. Moreover, it was also reported that non-antigenic PAA increases the immune response upon exposure with sheep erythrocytes, where they worked as an immunostimulants [17]. Thus, the prime objective of the current research was to synthesize the water-soluble ester prodrug of PPT with hydrophilic PAA and to examine its altered in vitro cell growth inhibition characteristics as a result of its improved solubility.

## Methods

### Materials and cell culture

Podophyllotoxin (PPT) was purchased from Shanghai Yuanye Bio-Technology Co., Ltd. (Shanghai, China). Polyacrylic acid (PAA), 4-(dimethylamino) pyridine (DMAP), and N, N′-dicyclohexylcarbodiimide (DCC), were procured from Sigma Aldrich, Mumbai, India. Dimethylformamide (DMF), HPLC grade acetonitrile, methanol, acetone, and water were procured from Molychem, Mumbai, India. All other analytical reagent grade chemicals were used as such without any further processing.

#### Cell culture

The cancerous MCF-7 and MDA MB-231(MDR epithelial human triple-negative) breast cancer cells, and non-cancerous human embryonic kidney cell line 293 (HEK-293) were obtained from ATCC, USA. Dulbecco’s modified eagle’s medium (DMEM) containing 10% FBS (fetal bovine serum), 100 IU/mL penicillin, and 100 μg/mL streptomycin was used to culture both cancerous and non-cancerous cells at 37 °C in a humidified atmosphere of 5% CO_2_.

#### Ethical approval

Ethical approval was not applicable.

### Synthesis of PPT-PAA conjugates (PPC)

**Fig. 1:**
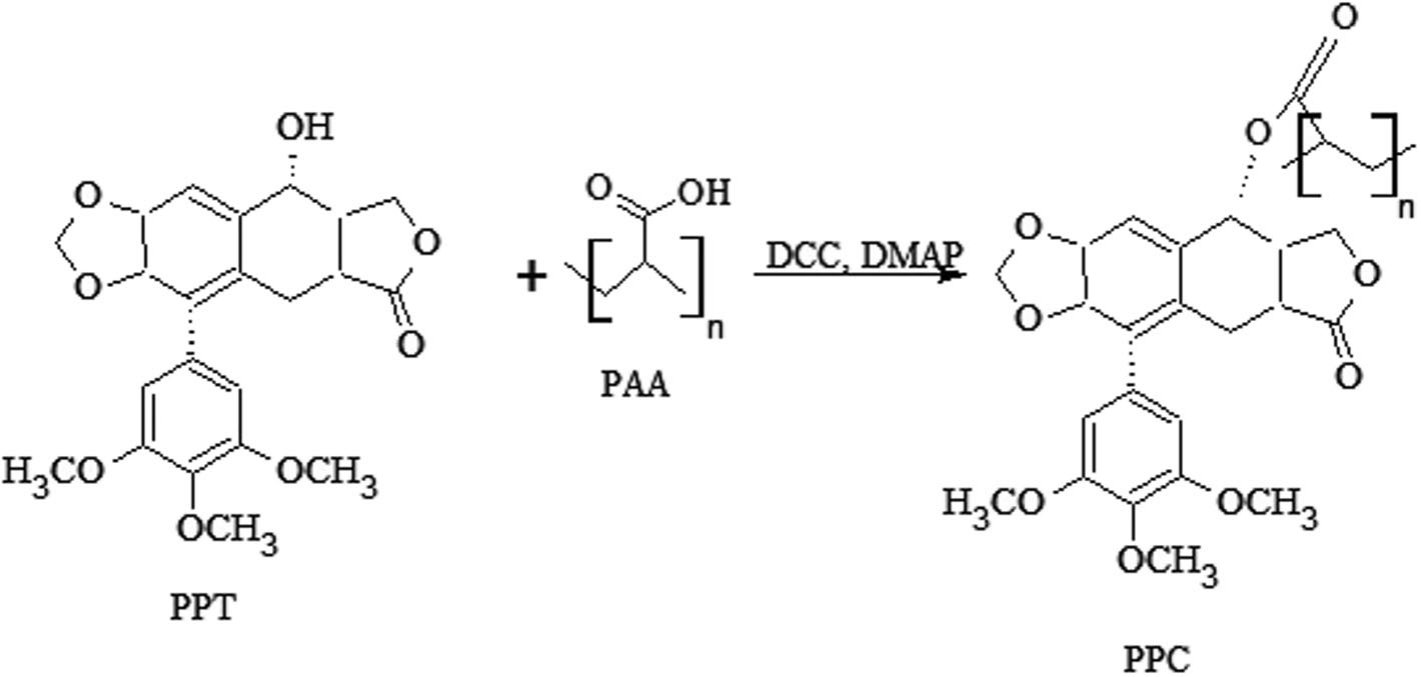
Synthetic route of PPC

The synthetic scheme of PPC is presented in Fig. 1. The PPT-PAA ester prodrug was synthesized at a 1:1 molar ratio using the Steglich esterification reaction according to previous reports. Briefly, PAA (0.0125 mmol, 25 mg) and DMAP (0.0250 mmol, 3.05 mg) were dissolved in DMF (5 mL) with stirring for 30 min. The DCC (0.0250 mmol, 5 mg) and PPT (0.0250 mmol, 10 mg) were then added under stirring. The reaction mixture was then kept at refrigeration (2 to 4 °C) for 10 min, and then for 24 h at room temperature under mild stirring. The precipitated side product dicyclo-hexyl urea (DHU) obtained was removed by filtration, and the filtrate was subjected to dialysis against double distilled water using a pre-activated dialysis bag of 12,000 molecular weight cutoff (12,000 MWCO) for 24 h. After that, the content present in the dialysis bag was isolated by filtration. The filtrate obtained was evaporated under reduced pressure in a rotary evaporator at 70 °C. The obtained product (residue) was then subjected to the confirmation using FTIR and NMR [18].

### Characterization of PPC

#### Spectroscopic characterization

##### FTIR analysis

The structure of PPC was analyzed by FTIR. The FTIR spectra of PPT and PPC were recorded using an Agilent FTIR spectrophotometer (Agilent, Alpha 100508) over the wavenumber 4000 to 400 cm^−1^.

##### NMR analysis

The ^1^H NMR analysis was also performed to confirm the structure of PPC. The ^1^H NMR spectra of the PPT and PPC were recorded by dissolving them in dimethyl sulphoxide (DMSO) using FT-NMR Bruker 300 Avance (300 MHz).

#### PPC assay

Briefly, 3 mg of PPC was dissolved in 3 mL of methanol to get a stock solution concentration of 1000 μg/mL. This solution was then allowed to sonicate for 5-10 min and finally analyzed by a UV-visible spectrophotometer at 290 nm against methanol as a blank solution [18].

#### Aqueous solubility

The aqueous solubility of PPC was studied by using the rotary shaker method. Briefly, the surplus amount of PPC was added into Eppendorf tube containing 0.5 mL of double distilled (DD) water and kept on a rotary shaker for 24 h. On completion of 24 h of shaking, the filtrate obtained by filtration was subjected for analysis (after suitable dilution with DD water) using a UV-visible spectrophotometer at 290 nm and the PPT content was determined [18].

#### In vitro hemolysis assay

The hemolysis test was performed in accordance with our previous report. Briefly, the erythrocyte pellet (RBCs) obtained by 10 mL of human blood (defibrinogenated) was diluted (with 0.9% NaCl solution) to obtain a 2% solution of RBCs (stored at refrigeration temperature till further use). The prepared PPT and PPC solutions were then added to the RBCs solution (1 mL), and the volume was adjusted to 5 mL with sodium chloride (NaCl) solution to obtain 10 and 100 μg/mL PPT and PPC solutions. The above solutions were then stored for 1 h at 37 °C. After incubation, the solutions were centrifuged (at 1000 rpm for 10 min), clear and transparent supernatant was collected and subjected for hemoglobin analysis using a UV-visible spectrophotometer at 420 nm. The positive and negative controls used were de-ionized water and NaCl solution (0.9%), respectively. The % hemolysis was calculated using the following formula [18, 19].

$$ \% Hemolysis=\frac{\mathrm{Test}\ \mathrm{sample}\ \mathrm{absorbance}-\mathrm{Negative}\ \mathrm{control}\ \mathrm{absorbance}}{\mathrm{Positive}\ \mathrm{control}\ \mathrm{absorbance}-\mathrm{Negative}\ \mathrm{control}\ \mathrm{absorbance}}\times 100 $$

#### In vitro cytotoxicity

The effect of PPT and PPC on the viability of cancerous cells (MCF-7 and MDAMB-231) and non-cancerous cell (HEK-293) was determined by using 3-(4, 5-dimethylthiazol-2-yl)-2, 5-diphenyltetrazolium bromide (MTT) dye reduction assay [18]. Briefly, 50,000 cells were transferred to each well of 96-well plates and allowed to adhere and grow. After overnight incubation in a 5% CO_2_ and 37 °C atmosphere, the supernatant DMEM was replaced with 100 μL of serially diluted test solutions (PPT and PPC). The 96-well plates were then incubated for 48 h in a similar environment. Post incubation, the supernatant drug solutions were replaced with the same volume of MTT stock solution prepared in phosphate buffer solution (PBS) 0.6 mg/mL. After 4 h incubation, the MTT solution was replaced with the same volume of DMSO that dissolves the formazan crystals formed in viable cells. The absorbance of the resultant DMSO solutions was measured at 590 nm using an ELISA plate reader. The absorbance of the test samples was compared to the absorbance of untreated cells to obtain the dose-response curves, and IC_50_ values were calculated from the same curves [18].

#### Critical micelle concentration (CMC)

The self-assembling of prepared PPC into micelles was determined by the iodine UV-visible spectrophotometric method. The double-distilled water was used to prepare PPC solutions of different concentrations. The prepared test solutions were mixed with a fixed volume (25 μL) of potassium iodide/iodine solution and incubated overnight (12 h) in dark at room temperature. Post incubation, the absorbance of test samples and blank sample (containing no PPC) were measured, against double distilled water as a blank, at 366 nm [20].

### Preparation of micelles

PPC micelles were prepared using a thin-film hydration technique. Briefly, 2.0 mg of PPC was dissolved in 2 mL of methanol in a beaker. The PPC film was obtained by evaporating methanol at room temperature. The PPC film was redispersed using a fixed volume of double-distilled (10 mL) and using a bath sonication technique. Post redispersion, the micellar solution was centrifuged (5000 rpm) to obtain a clear solution free from visible dispersed particles [18].

### Characterization of micelles

#### Mean particle size

The mean particle size and zeta potential of PPC micelles were investigated using Malvern ZS Zetasizer (Malvern Instruments Ltd.) at room temperature (25 °C) and measurements were carried out in triplicate.

#### Morphology by TEM

In brief, the test sample (2 drops of micellar solution) was transferred on to a copper grid (nitrocellulose covered) and air-dried for 12 h at room temperature. Then, the dried test sample was stained using a 2% w/v solution of phosphotungustic acid (negative staining). The sample was then air-dried at room temperature and observed under TEM (TEM, FEI Tecnai T-20ST).

#### In vitro release study

The dialysis tube technique was used to determine the in vitro release profile of PPT from the prepared PPC micelles [20]. The results were compared with the release profile of PPT from its dispersion prepared in PBS. Into dialysis tubes (MWCO = 12000 Da), the PPT-loaded PPC micelles and PPT dispersions equivalent to 2 mg of PPT were transferred. The tubes were then placed into beakers containing PBS (80 mL) of pH 7.4 as release medium. The release medium was maintained at 37 ± 2 °C and stirred (150 rpm) during the study. One milliliter of release medium was removed at a predetermined time (1, 2, 4, 8, 16, 24, and 48 h) for analysis. At the same time, a 1 mL fresh release medium was transferred back to the beakers. The test samples withdrawn were analyzed, following centrifugation and suitable dilutions with release medium, at 290 nm using a UV-visible spectrophotometer. The release profile of PPT from each formulation was carried out in triplicate. Cumulative % PPT release was calculated and plotted against time [12].

### Statistical analysis

Data are mentioned as the mean ± standard deviation of three independent experiments. The statistical analysis was performed by using the GraphPad Prism software version 5 (GraphPad Software, Inc., La Jolla, CA, USA). The results obtained were analyzed by one-way ANOVA. *p* < 0.05 was considered statistically significant.

## Results

### Synthesis and conformation of PPC

**Fig. 2:**
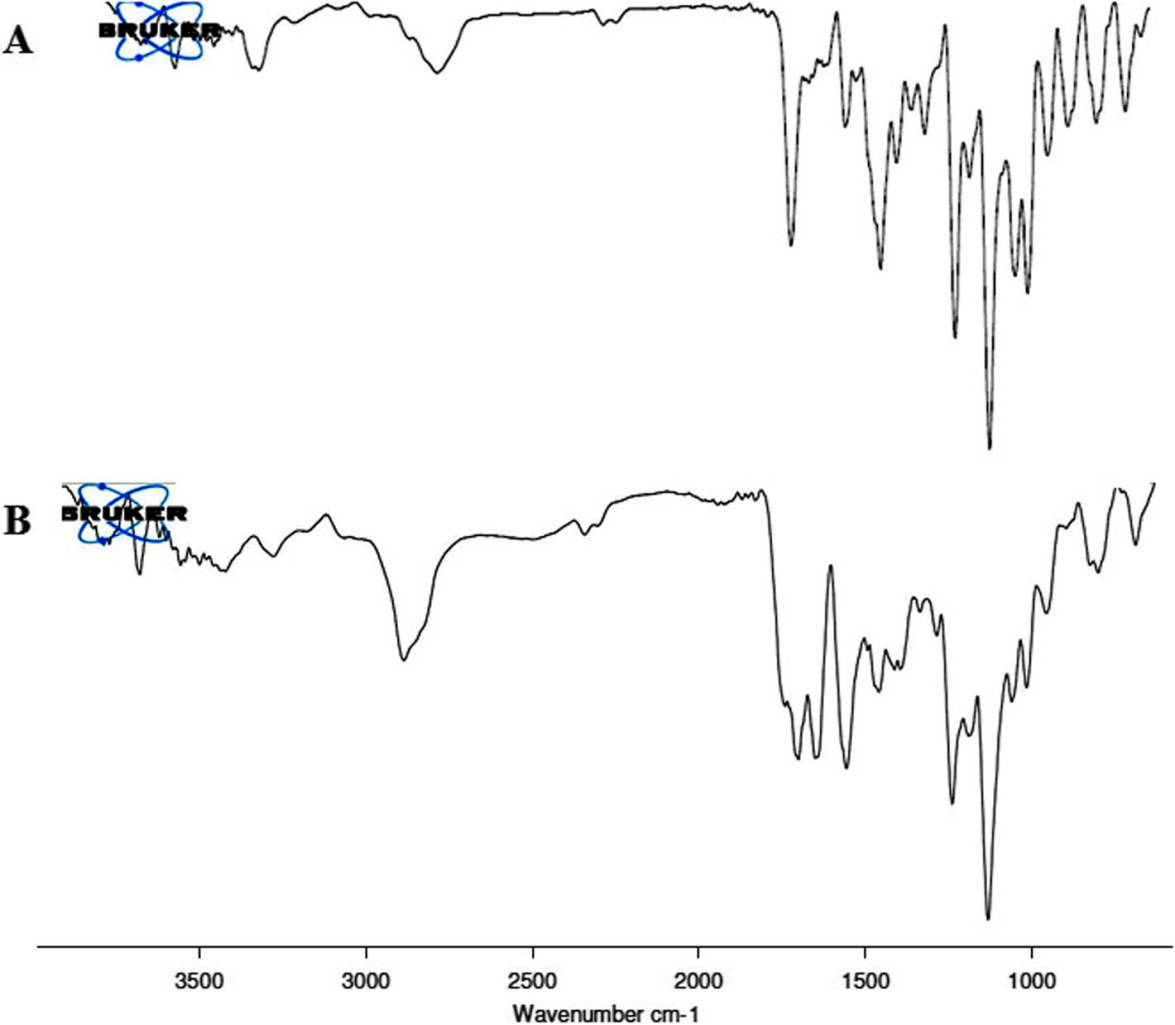
FTIR spectrum of (**a**) PPT and (**b**) PPC

The Steglich esterification reaction was used for the synthesis of PPC. The structure of the PPT prodrug is initially confirmed by FTIR. The FTIR spectra of PPT and PPC are shown in Fig. 2. In the FTIR spectrum of PPT (Fig. 2a), the peak at 2923 cm^−1^ is the characteristic peak of the benzene ring in PPT. In the FTIR spectrum of PPC (Fig. 2b), the peak at 1116 cm^−1^ is attributed to the C-O stretching vibrations of PAA while the peak at 1735 cm^−1^ indicates ester group formation with PPT. The obtained result demonstrated that PPC was synthesized successfully.

**Fig. 3:**
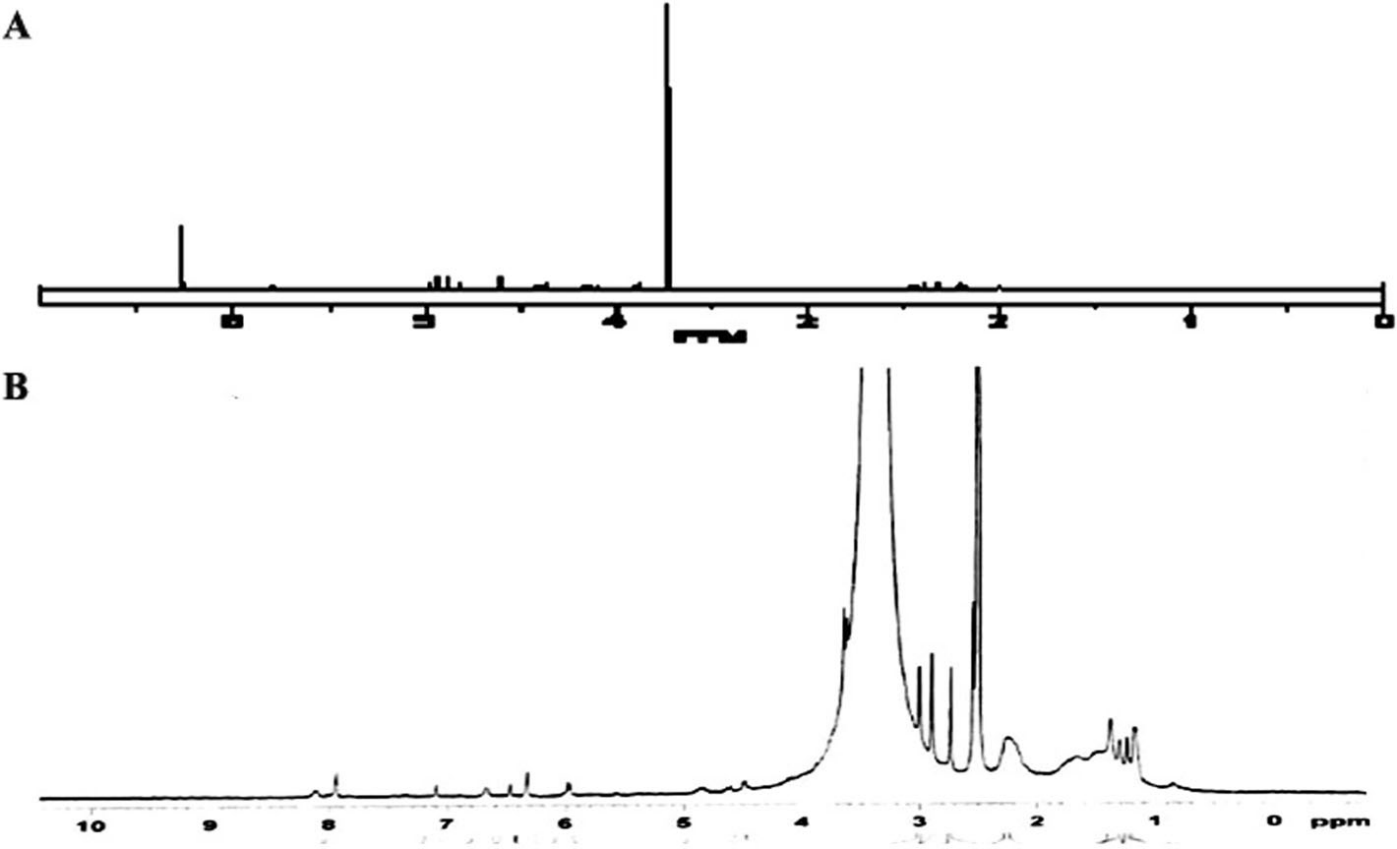
NMR spectrum of (**a**) PPT and (**b**) PPC

The structure of PPC is also confirmed with ^1^H NMR (Fig. 3). The peaks at 5.8-7.0 ppm are assigned to the phenyl groups of PPT (Fig. 3a). In the ^1^H NMR spectrum of PPC (Fig. 3b), the peaks at 5.5-7.7 ppm are characteristic peaks of the phenyl groups of PPT. Besides, we observed the chemical shift value of 3.2 ppm corresponding to the formation of an ester bond between PPT and PAA. This result also confirmed the successful synthesis of PPC. Further, these results are in accordance with the earlier report [21].

### Characterization of PPC

The assay of PPC was found to be 64.01 ± 4.5%. The solubility of PPC in water is found to be 1.39 ± 0.05 mg/mL (PPT equivalent) [12].

### In vitro hemolysis assay

The positive control (de-ionized water) showed complete (100%) hemolysis while very slight or no hemolysis (0%) was observed with the negative control (0.9% NaCl). The plain PPT displayed less hemolysis (12.8 ± 1.7%) at 10 μg/mL concentrations and more hemolysis (51.7 ± 6.4%) at 100 μg/mL. In contrast, PPC exhibited significantly (*p* < 0.01) less hemolysis (6.2 ± 1.2% and 18.6 ± 2.9%) than plain PPT at the same concentration.

### In vitro cytotoxicity

The antitumor activity of plain PPT and PPC was examined using MTT assay against cancerous cells (MCF-7 and MDAMB-231cell), and non-cancerous cell (HEK-293). The cytotoxicity of both PPT and PPC is expressed in terms of IC_50_ value. The IC_50_ value is the concentration of drug necessary to kill 50% of incubated cells in a designated time. The IC_50_ values of both PPT and PPC are depicted in Table 1. We observed substantially increased cytotoxicity (*p* < 0.01) with PPC (low IC_50_ values), against both cell lines, in comparison to plain PPT after 48 h study. On the other hand, both PPT and PPC showed significantly low cytotoxicity (*p* < 0.01) against non-cancerous (HEK-293) cells after 48 h of incubation when compared to cancerous cells.

**Table 1:** Comparison of IC_50_ values (μg/mL) of tested substances against breast cancer and non-cancerous cell lines

Cell lines	IC_**50**_ values (μg/mL)	
	48 h	
	Plain PPT	PPC
MCF-7	1.10 ± 0.18	0.608 ± 0.05
MDA MB-231	0.452 ± 0.06	0.146 ± 0.02
HEK-293	9.8 ± 0.56	2.1 ± 0.26

Values are mean ± SD (*n* = 3)

### CMC of PPC micelles

The CMC of PPC micelles was found to be 0.430 mg/mL.

### Preparation and characterization of PPC micelles

**Fig. 4:**
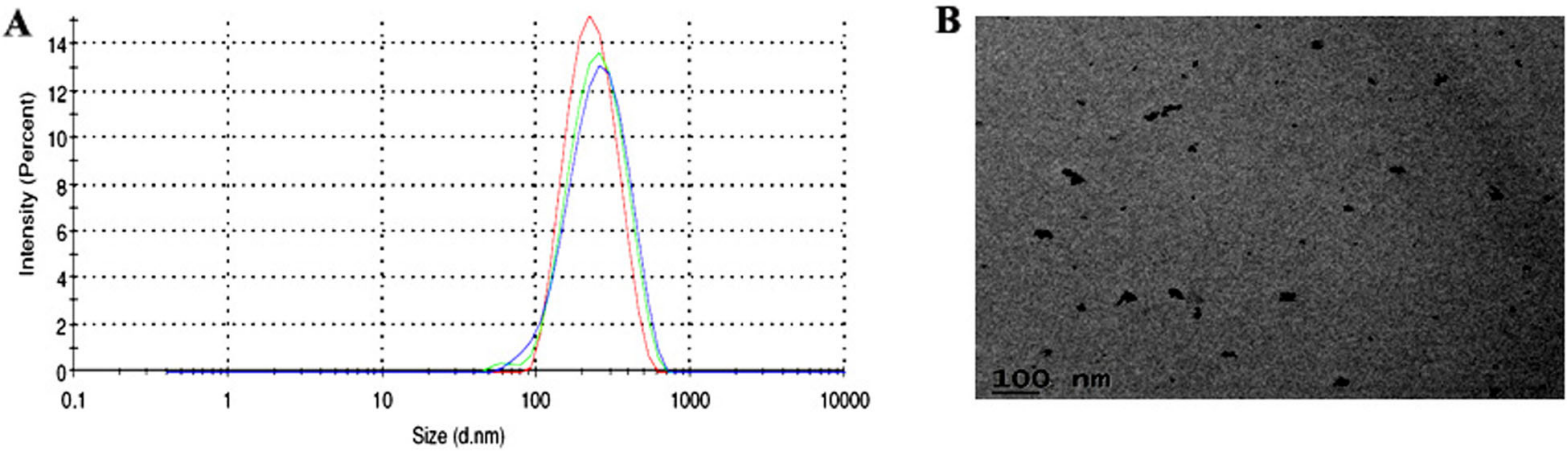
The mean particle size (**a**) PPC micelles and (**b**) TEM image showing self-assembling nature of PPC

The mean particle size of PPC micelles measured by Malvern zeta sizer is found to be 215 ± 11 nm with PDI of 0.193 ± 0.006 (Fig. 4a). The zeta potential of PPC micelles was found to be −37.3 mV. Furthermore, the structure of PPC micelles was confirmed by TEM analysis. The TEM image of self-assembled PPC micelles (Fig. 4b) showed that the micelles are well dispersed and spherical in shape.

### In vitro release study

**Fig. 5:**
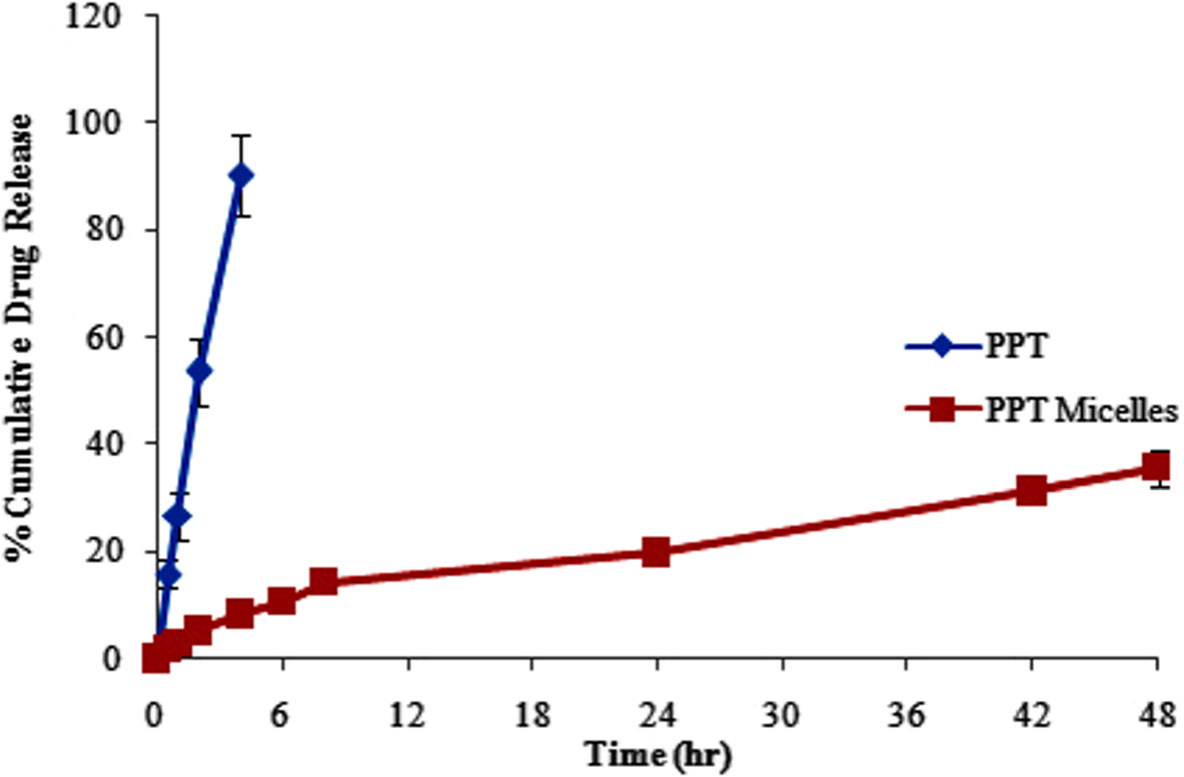
Cumulative % of PPT released from the plain PPT solution and PPC micelles

The release profile of PPT from plain PPT solution and PPC micelles is depicted in Fig. 5. Above 90% of PPT is released from the PPT dispersion in just 4 h. On the other hand, PPT release from PPC micelles is found significantly (*p* < 0.01) sustained (35 ± 3.2% after 48 h of the study) in comparison to PPT dispersion.

## Discussion

In the current research, the PPC is synthesized via the ester bond formed between the reactive C-4 hydroxyl group of PPT and the carboxyl groups of the hydrophilic polymer, PAA [13, 14]. The solubility of PPT is found to be about 200-fold higher than the reported standard PPT solubility (0.001 mg/mL) following conjugation. Thus, the aqueous solubility of PPT following conjugation with PAA is found to increase substantially. The increased solubility of PPT is due to its conjugation with hydrophilic PAA.

The chief prerequisite of any type of nanomedicine is its good compatibility with blood. Plain PPT demonstrated significantly more hemolysis (3.0-fold increased hemolysis) than PPC. The hemolysis test results clearly revealed low or moderate hemolysis by the PPC showing good biocompatibility. Thus, the obtained results suggest that PPC micelles are safe and biocompatible for intravenous injection.

We found 2.0-fold and 3.0-fold higher cytotoxicity of PPC than plain PPT against MCF-7 and MDA MB-231 cells respectively. The significantly enhanced cytotoxic activity of PPC against cancerous cells might be due to endocytosis and increased intracellular accumulation of PPT by nanoparticle uptake. Moreover, the nanometer particle size of PPC micelles and sustained release of PPT from PPC micelles also responsible for the higher cytotoxic activity of PPC. Further, the above results are in accordance with the earlier report [22].

To test the toxicity of PPT and PPC toward healthy cells, in the present study, we also performed the in vitro cytotoxicity study against HEK-293 cells. The obtained results revealed the less cytotoxicity (significantly higher IC_50_ values) of PPT and PPC toward non-cancerous HEK-293 cells as compared to cancerous cells.

The CMC value is a chief parameter used to evaluate the micelle forming ability of block copolymer. It can also reflect the stability of the micelles upon in vivo dilutions. The PPC micelles showed significantly less CMC. These relatively lower CMC of PPC micelles revealed that they would remain stable in solutions even after in vivo dilution [23–25].

The higher mean particle size of PPC micelles could be due to the presence of more PPT-PAA conjugates, which can enlarge the micelles inner hydrophobic core. The PDI value near to zero of PPC micelles suggested the uniform size and homogeneity of the micelles.

The higher release rate of PPT from PPT dispersion could be attributed to its free diffusion through the dialysis membrane into a release medium. PPC micelles demonstrated the sustained release of PPT that could prolong the circulation time and minimize the exposure of PPT to healthy tissues. Furthermore, this behavior could also increase the accumulation of PPT in tumors via the EPR effect and increase the therapeutic efficacy.

## Conclusion

In the current investigation, the water-soluble prodrug of PPT was synthesized by conjugating it with the hydrophilic PAA polymer. The prodrug demonstrated improved water solubility and substantial in vitro cell growth inhibition characteristics. Besides, it demonstrated lower hemolytic behavior which stipulates its appropriateness for intravenous applications. The marvelous in vitro cytotoxic behavior of prodrug against MDR breast cancer cells indicates its application in the treatment of MDR tumors. The prodrug is found to be self-assembled into micelles with low CMC value indicating its high stability upon systemic dilution. In addition, prodrug micelles exhibited sustained drug release which could cause increased circulation half-life and reduced systemic toxicity. Thus, the current approach could be promising for the efficient treatment of MDR cancers. However, further in vivo studies are needed to validate these obtained in vitro results.

## Data Availability

All data and materials are available upon request.

## Abbreviations

BCS: Biopharmaceutical classification system
CMC: Critical micelle concentration
DCC: N, N′-dicyclohexylcarbodiimide
DHU: Dicyclo-hexyl urea
DMAP: Dimethylamino pyridine
DMEM: Dulbecco’s modified eagle’s medium
DMF: Dimethyl formamide
DMSO: Dimethyl sulphoxide
EPR: Enhanced permeation and retention effect
FBS: Fetal bovine serum
FTIR: Fourier transform infrared
MDR: Multidrug resistance
MTT: 3-(4, 5-dimethylthiazol-2-yl)-2, 5-diphenyltetrazolium bromide
MWCO: Molecular weight cutoff
NMR: Nuclear magnetic resonance
NaCl: Sodium chloride
PAA: Polyacrylic acid
PBS: Phosphate buffer solution
PDI: Polydispersity index
P-gp: P-glycoprotein
PPC: Podophyllotoxin-polyacrylic acid conjugate
PPT: Podophyllotoxin
TEM: Transmission electron microscope
